# The Unfinished Debate on Wine and Other Alcoholic Beverages: Conflicting Evidence, Public Health Messages and the Missing Trial

**DOI:** 10.3390/nu18030529

**Published:** 2026-02-05

**Authors:** Miguel Angel Alvarez-Mon, Diego Martínez-Urbistondo, María Barbería-Latasa, Zenaida Vázquez-Ruiz, Miguel Ruiz-Canela, Maira Bes-Rastrollo, Miguel Ángel Martínez-González

**Affiliations:** 1 Department of Psychiatry, Infanta Leonor University Hospital, 28031 Madrid, Spain; 2 CIBERSAM-ISCIII (Biomedical Research Networking Centre in Mental Health), 28029 Madrid, Spain; 3 Department of Legal and Psychiatry, Complutense University, 28040 Madrid, Spain; 4 Ramón y Cajal Institute of Sanitary Research, 28034 Madrid, Spain; 5 Vascular Medicine Area, Clínica Universidad de Navarra, 28027 Madrid, Spain; dmurbistondo@unav.es; 6 Department of Internal Medicine, Clínica Universidad de Navarra, 28027 Madrid, Spain; 7 CIBER Fisiopatología de la Obesidad y Nutrición (CIBERobn), Instituto de Salud Carlos III, 28029 Madrid, Spain; zvazquez@unav.es (Z.V.-R.); mcanela@unav.es (M.R.-C.); mbes@unav.es (M.B.-R.); mamartinez@unav.es (M.Á.M.-G.); 8 Department of Preventive Medicine and Public Health, Faculty of Medicine, Institute for Nutrition and Health, University of Navarra, 31008 Pamplona, Navarra, Spain; mbarberia.3@unav.es; 9 IdiSNA, Navarra Institute for Health Research, 31008 Pamplona, Spain; 10 Department of Nutrition, Harvard T.H. Chan School of Public Health, Boston, MA 02115, USA

**Keywords:** alcohol consumption, public health policy, Mendelian randomization, observational studies, randomized controlled trials

## Abstract

The overall health impact of alcohol remains controversial. The Global Burden of Disease (GBD) study made headlines in 2018 by stating that zero alcohol was the safest option. However, its 2022 update introduced age-specific adaptations, asserting that moderate drinking may offer net benefits in some populations. The 2025 U.S. Surgeon General report also discouraged any alcohol intake because of associated cancer risks, but a simultaneous report by the National Academies emphasized tailoring recommendations to individual risks. Mendelian randomization (MR) studies found no health benefits and highlighted increased risks—even at low alcohol doses—challenging dozens of conventional epidemiologic findings in large observational cohorts, some of them of high methodological quality. Given these contradictions, there is a pressing need for large randomized controlled trials in drinkers promoting cessation versus moderation. While small trials have shown some metabolic and cardiovascular benefits of moderate red wine consumption, no large-scale randomized trial has yet assessed hard clinical outcomes comprehensively. Without such evidence, debates will persist. Current guidelines appropriately discourage alcohol in certain populations, but generalizations such as “no safe level of alcohol” might lack sufficient empirical support and perhaps they need a tailored and practical strategy in the context of precision medicine. A balanced, evidence-based approach—free from bias, independent of the industry and grounded in rigorous research—is essential for informed public health policy decisions.

## 1. Zero, Moderate, or It Depends? The Unfinished Debate on Alcohol and Risk

The relationship between alcohol consumption and health has long been a subject of scientific discussion and public interest. In recent years, this topic has become increasingly controversial. A pivotal moment came in 2018, when the Global Burden of Disease (GBD) study—one of the world’s most comprehensive efforts to quantify global causes of morbidity and mortality—published a landmark paper in The Lancet [1]. The authors concluded unequivocally that “the level of alcohol consumption that minimizes health loss is zero.” This sweeping assertion, grounded in data from 195 countries, garnered intense academic and media attention. It suggested that even small amounts of alcohol posed risks that outweighed any potential benefits, challenging previous assumptions about light-to-moderate drinking [1]. However, this stark conclusion did not remain unchallenged for long. In 2022, the same GBD group revised its stance in a new report with new added data collected for the period 2016–2020. This updated report was also published in The Lancet [2]. While maintaining concerns about the many risks associated with alcohol consumption, the 2022 GBD report introduced an age-specific, risk-weighted model. In populations with high baseline rates of cardiovascular disease—particularly among older adults—light-to-moderate alcohol consumption could be associated with net health benefits, particularly when a high background ischemic cardiovascular risk is present [2]. The revised conclusion did not reverse the earlier findings entirely but highlighted the need for more context-specific recommendations. One-size-fits-all guidelines may not reflect the reality of alcohol’s heterogeneous effects [2].

The scientific tension escalated further in January 2025 with the release of two high-profile U.S. reports. On 3 January 2025, the Surgeon General, then Dr. Vivek Murthy—the country’s top public health official—issued an Advisory report on Alcohol and Cancer [3]. That report emphasized that even low levels of alcohol intake increase the risk of at least seven types of cancer, estimating that alcohol accounts for over 100,000 cancer cases and 20,000 cancer deaths annually in the United States. The Advisory report called for enhanced public awareness and updated warning labels on alcoholic beverages, advocating a strong, precautionary stance. In the same month, the National Academies of Sciences, Engineering, and Medicine released the “Review of Evidence on Alcohol and Health,” commissioned by the Congress to inform the upcoming Dietary Guidelines for Americans (2025–2030) [4]. This comprehensive review acknowledged both the potential cardiovascular benefits of light-to-moderate drinking and the elevated risks for cancer and other health conditions. Importantly, the report did not call for universal abstention, instead, it highlighted the complexity of the evidence and the importance of tailoring recommendations to individual risk profiles [4]. Subsequently, the Academies’ report received criticisms, arguing that it downplayed the alcohol risks even at low levels of intake, by omitting key studies on cancer, cognitive decline, and cardiovascular disease. Doubts also emerged on the overlooking of recent genetic studies that challenged the alleged benefits of light-to-moderate drinking [5]. The finally issued DGA 2026–2030 stated that adults who choose to drink alcohol should “consume less alcohol for better overall health”, but no quantitative upper limit was mentioned.

Together, these recent documents illustrate the evolving—and at times contradictory—landscape of the scientific understanding of the effects of light-to-moderate alcohol intake on health. Evidence is both vast and conflicting, and interpreting it requires comprehensive and context-sensitive insights adapted to the population characteristics, types of alcoholic beverages consumed, drinking patterns, and the endpoints being analyzed in the context of precision and personalized medicine. The following is an overview of influential studies on the matter.

Relevant studies were identified for this Viewpoint through targeted searches of PubMed and other databases using terms related to alcohol consumption, health outcomes, Mendelian randomization, cohort studies, randomized trials and drinking patterns. High-impact publications from the past three decades (1995–2025) were prioritized, while earlier influential studies were included where appropriate. The evidence was synthesized qualitatively to highlight key findings, methodological differences, and areas of research requiring further investigation.

## 2. Evidence from Cohort and Mendelian Randomization Studies: Quantity vs. Drinking Pattern

A large cohort study examined the health effects of alcohol consumption in over half a million Chinese adults, with a particular focus on men, who represented the overwhelming majority of drinkers in China [6]. Drawing on data from the China Kadoorie Biobank, researchers followed participants for an average of 12 years and investigated the relationship between alcohol intake and 207 different diseases using both conventional epidemiological analyses and Mendelian randomization [6]. They reported that alcohol consumption in Chinese men was associated with significantly higher risks of 61 diseases, including 33 not previously recognized by the World Health Organization as alcohol-related. Notably, the study found no evidence of health benefits from alcohol at any level of consumption. While earlier studies—mostly from Western populations—have reported apparent protective effects of light-to-moderate drinking for certain cardiovascular conditions, this study suggested that such associations are likely due to biases such as residual confounding and reverse causation. It should be noted that the study did not distinguish health risks by specific types of alcoholic beverages, and alcohol intake was assessed via self-reported questionnaires, with no use of objective biomarkers or direct measurements.

A more recent study explored the complex relationship between alcohol consumption and ischemic heart disease (IHD), applying an advanced “burden of proof” meta-analytic framework to integrate evidence from cohort studies, case–control studies, and Mendelian randomization (MR) studies [7]. While traditional observational studies suggested that light-to-moderate alcohol intake may reduce the risk of IHD and myocardial infarction—particularly in men—these findings may be skewed by biases such as the “sick quitter” effect and misclassification of drinking habits. In contrast, MR studies, which use genetic proxies to infer causality, found no protection or even slightly higher risks, casting doubt on previous assumptions. The study also considered how the drinking patterns influence outcomes: binge drinking raised IHD risk, while light-to-moderate intake may appeared protective in some analyses, though this was not supported by MR findings [7]. By adjusting for multiple sources of bias, the “burden of proof” approach revealed a significant discrepancy between observational and MR studies. The authors argued that more rigorous methodologies—such as emulated trials within large observational datasets—are needed to clarify the true impact of alcohol on cardiovascular health and inform public health guidance more accurately. Very importantly, MR studies, which are valid only under certain assumptions, cannot assess the drinking pattern.

Along the same lines, a study published in 2024 using a multivariable Mendelian randomization design to investigate the association between alcohol consumption and 52 types of cancer—using genetic data from over 150,000 individuals in the UK Biobank—reported that higher alcohol intake was associated with an increased risk of several cancers, including those of the mouth, esophagus, liver, colon, rectum, and breast [8]. These findings suggest that ethanol itself appears to be the main culprit, regardless of the type of beverage, and reinforce the evidence that alcohol is a carcinogen, thus confirming the long-held position of the International Agency for Research on Cancer (IARC), which classified alcoholic beverages as carcinogenic to humans (Group 1) on the basis of sufficient evidence of causality.

The SUN cohort reported in 2014 that low-to-moderate alcohol consumption, particularly when integrated into a Mediterranean Alcohol Drinking Pattern (MADP)—characterized by preference for wine, drinking with meals, avoidance of binge drinking and spreading the consumption along the full week—was associated with reduced all-cause mortality keeping constant the total amount of ethanol consumed [9]. Limitations include a relatively homogenous and highly educated cohort, which may affect generalizability. Subsequently, other observational studies also partially replicated these findings on the modifying effect by the drinking pattern. In 2021, Jani et al. analyzed more than 300,000 regular alcohol consumers in the UK Biobank to investigate how patterns of alcohol use—specifically beverage type, frequency, and context of drinking—may influence the risk of adverse health outcomes [10]. Over a median follow-up of nine years, they found that participants who primarily drank red wine, consumed alcohol with meals, and spread their intake over 3–4 days per week had lower risks of mortality, cardiovascular events, and liver cirrhosis [10]. In contrast, drinking spirits or beer instead of wine, consuming alcohol with an empty stomach, or drinking only once or twice a week was associated with higher risks, even when total ethanol intake was the same. Regarding plausible biological mechanisms, a large study on different alcoholic beverages and cardiovascular risk biomarkers reported differential effects on inflammatory biomarkers by the different alcoholic beverages [11]. Particularly, red wine exhibited the strongest anti-inflammatory effects whereas hard liquors were associated with higher interleukin-6 levels, yet equally associated with lower HbA1c and higher HDL-cholesterol as other beverages. Other potential beneficial aspects of the MADP were also in the expected direction of the beneficial effect modification by this drinking pattern on the effect of alcohol [11].

Together, these studies offer compelling evidence that drinking patterns—including the type of alcohol, frequency, and drinking context—might play critical roles in determining alcohol-related health risks. While each study varies in scope and methodology, they converge on a key insight: the absolute amount of consumed ethanol may not be the most relevant exposure because not all patterns of light-to-moderate drinking would be equal in terms of health effects. This shared conclusion challenges traditional public health advice that focused solely on the total amount of ethanol intake. But how and when people drink may be just as important as how much they drink. The total amount of ethanol consumed may not be the most relevant factor, as the ‘quality’ of the drinking pattern appears to be important. This is similar to debates in nutrition, where the quality of carbohydrates and fats matters more than the quantity.

In any case, it is worth noting that the largest cohort study so far to our knowledge was conducted in the UK. Investigators took care to separate former drinkers, so as to avoid the so-called “sick quitter” effect and they reported that the lowest adjusted all-cause mortality corresponded to moderate alcohol consumers after analyzing for 6 years 1.93 million adults in England who were initially free from cardiovascular disease [12]. All-cause mortality is a very important end-point because it may represent a balanced summary of harmful effects on cancer and potential protection on cardiovascular ischemic events and diabetes. In fact, in this British CALIBER cohort, moderate alcohol intake was associated with a reduced risk of myocardial infarction, angina, heart failure, and ischemic stroke [12]. However, it was also linked to an increased risk of other cardiovascular outcomes, such as hemorrhagic stroke and cardiomyopathy. In another cohort of over 900,000 adults, a J-shaped relationship between alcohol use and mortality was also observed, in consistency with many previous conventional cohort studies [13]. Moderate consumption may offer some protective effects according to the reports by these conventional cohorts, but several potential biases cannot be excluded, such as reverse causation, incomplete control for confounding, selection bias, misclassification, or the above-mentioned “sick-quitter” effect. These results demonstrate alcohol’s outcome-specific risk profiles, challenging uniform consumption guidelines. In any case, and without any hesitation, heavy or binge drinking and drinking in the youth is clearly harmful. Public health recommendations should consider both the potential benefits and the risks associated with alcohol use [13]. Nevertheless, most of the discussed evidence originates from high-income Western populations. The implications for low-and middle-income countries, whose drinking patterns and disease burdens differ, have not been sufficiently explored. While the China Kadoorie Biobank study provides valuable data from a large middle-income population, further research is needed in diverse global contexts [6].

## 3. Moderate Wine Consumption Within the Mediterranean Diet

The Greek-EPIC cohort was the first large prospective study, with good control for confounding, evaluating an operationalized definition of the Mediterranean diet. Its report published more than 20 years ago, showed a strong inverse association between adherence to the Mediterranean diet and all-cause mortality [14]. For each two-point increase in the 0-to-9 Mediterranean diet score, there was a 25% reduction in the risk of all-cause mortality. But perhaps most interestingly, the component of this diet most strongly associated with lower mortality was moderate alcohol consumption [14]. This means that when moderate alcohol intake was removed from the operational definition of the Mediterranean diet a sizable part of its protection was lost. Consequently, both the PREDIMED randomized trial and the CORDIOPREV randomized trial reporting an approximate 30% lower incidence of major cardiovascular events with interventions using the Mediterranean diet included moderate alcohol intake as part of these beneficial interventions [15,16]. Among the 14 dietary recommendations given to participants randomized to the Mediterranean diet groups, moderate red wine consumption with meals was included in both trials.

The consistent inclusion of moderate wine consumption in operational definitions of the Mediterranean diet, across observational cohorts (e.g., Greek EPIC) and RCT (e.g., PREDIMED, CORDIOPREV), may reflect confirmation bias among researchers familiar with traditional Mediterranean dietary patterns [14,15]. This cultural presupposition could influence study design, potentially resulting in the overlooking of alternative dietary configurations that are equally protective but wine-free. However, the biological plausibility of the effects of wine’s polyphenols, supported by biomarker studies, lends credence to these findings, which are not merely the result of cultural bias [17,18]. Although confirmation bias is a methodological concern, the convergence of RCT, cohorts, and biomarkers strengthens the observed association between the Mediterranean diet and wine consumption.

## 4. Randomized Trials and Objective Biomarkers: The Evidence Still Needed

In this context, there is a clear need for a randomized controlled trial to address this issue. In non-randomized studies, insufficient control of smoking—often due to suboptimal measurement granularity—may lead to misattribution of smoking-related health risks to alcohol, since drinkers typically show higher tobacco exposure than abstainers. Mendelian randomization studies, while valuable, are also subject to potential threats such as residual confounding, genetic pleiotropy, adaptation, weak instrument bias, and lack of replication. Moreover, drinking patterns—likely important effect modifiers—have been largely overlooked, both in MR studies and in Global Burden of Disease (GBD) analyses, because their focus was only the quantity of ethanol consumed and not the type of beverage or the drinking pattern. Conventional observational studies also face well-known methodological issues as already mentioned.

Another limitation of previous studies is the absence of objective markers of alcohol consumption. Although multiple classes of biomarkers are associated with alcohol consumption, relatively few beverage-type–specific biomarkers have been explored. In this regard, a recent case–cohort study examined the relationship between moderate wine consumption and cardiovascular health using urinary tartaric acid as an objective and highly specific biomarker of wine intake. Moderate levels of urinary tartaric acid (reflecting 12 to 35 wine glasses per month) were associated with the lowest cardiovascular risk (50% relative reduction), supporting the idea that wine, consumed moderately within a Mediterranean dietary context, may offer cardiovascular protection [18]. Unlike many previous studies based on self-reported intake, these conclusions are strengthened by the use of objective intake biomarkers, offering more robust evidence of the physiological impact of moderate wine consumption.

The largest randomized trial conducted to date on alcohol was the CArdiovaSCulAr Diabetes & Ethanol (CASCADE) study, which lasted two years and included 224 well-controlled type 2 diabetes patients [19]. All participants were initially abstainers, and those randomized to begin drinking red wine showed increases in HDL-C and Apo A1, as well as a reduction in the number of metabolic syndrome components compared to the mineral water group. In Italy, another RCT involving 131 patients with myocardial infarction and diabetes, randomized to a Mediterranean-style diet with or without the addition of red wine, found that the red wine group had higher HDL levels, lower oxidative markers, reductions in multiple inflammatory biomarkers, lower fasting insulin levels, and improved left ventricular function after one year [20]. In 2020, a 6-month RCT involving 140 drinkers across six hospitals in Australia concluded that alcohol cessation/reduction (without any specification of the type of beverage) reduced atrial fibrillation (AF) recurrences in patients with prior AF who were in sinus rhythm at baseline [21]. Surprisingly, apart from these 140 patients with a previous AF, no other RCT to date has evaluated the risk of any major “hard” clinical outcome in drinkers comparing two alternative alcohol interventions (cessation versus maintaining only light-to-moderate consumption as a harm-reduction approach). A previous attempt to conduct such a trial—the MACH15 study, which aimed to assess cardiovascular events—was well-designed and carefully planned, but was ultimately canceled by the U.S. National Institutes of Health due to concerns about potential conflicts of interest [22,23].

The IARC recently published a review on the available scientific evidence supporting the effects of alcohol cessation on cancer risk [24]. Beyond confirming that alcohol consumption was a clear risk factor for multiple types of cancer, only in two cancers of the upper digestive tract (mouth and esophagus) there was sufficient evidence that the risk progressively decreased after alcohol cessation, especially after 5–10 years of abstinence. However, for other cancers, such as breast cancer, the reduction in risk following alcohol cessation remains unclear.

Without any really new substantial evidence, scientific societies around the world are issuing or updating statements on alcohol policies, without reaching a completely consistent position. The American Heart Association published a statement in 2025 after reviewing the available scientific evidence on alcohol, suggesting that the cardiovascular benefits previously attributed to light-to-moderate alcohol consumption may actually be due to residual confounding in conventional epidemiologic studies [25]. However, previous small trials—reasonably free of confounding—and studies based on cardiovascular biomarkers did show benefits [18,19,20].

Unless a large-scale randomized controlled trial is conducted to provide high-level scientific evidence, alcohol-related debates based on non-randomized studies and only small trials will remain inconclusive and ongoing for decades [17]. Sound guidance for personalized clinical practice and individual-adapted public health guidelines is needed in the era of precision medicine. Robust evidence is urgently needed to issue such guidelines and to support clinical practice. Future trials should assess the effects of recommending to adult drinkers moderation versus cessation, using a comprehensive, holistic endpoint including not only cardiovascular disease, but also cancer, other chronic diseases, serious injuries, major infectious diseases, and all-cause mortality. It is crucial to appropriately weigh potential benefits and harms, starting from an agnostic perspective free from any conflict of interest.

Alcohol exhibits disease-specific risk curves. J-shaped patterns have been reported for ischemic heart disease, type 2 diabetes, and all-cause mortality in some observational cohorts. In contrast, monotonically increasing risks are consistently observed for cancers and certain types of strokes. However, MR and other bias-adjusted analyses often find no protective effects, or even slightly increased risks, at low levels across outcomes. This uncertainty highlights the need for definitive RCT before formulating categorical public health recommendations. Until such evidence emerges, precision medicine approaches tailored by age, sex, genetics and comorbidity may be a more appropriate framework than universal abstinence messaging.

At present, there is sufficient evidence to discourage any alcohol consumption in certain populations—for example, during pregnancy, those with medical conditions worsened by alcohol, or those taking medications that contraindicate any alcohol use. However, there is probably not enough evidence to support sweeping statements such as “there is no safe level of alcohol consumption,” as declared by respected institutions like the World Health Organization, or to fully support blanket ‘no safe level’ recommendations, even when issued by authoritative bodies like the IARC Working Group [26,27]. This type of recommendations should be framed in the context of precision medicine. Clinicians and public health agencies are responsible for ensuring that guidance on alcohol use is proportionate to the available evidence and tailored to individual risk profiles.

## Data Availability

Not applicable.
